# Active Suppression Induced by Repetitive Self-Epitopes Protects against EAE Development

**DOI:** 10.1371/journal.pone.0064888

**Published:** 2013-05-30

**Authors:** Fabiola Puentes, Katharina Dickhaut, Maria Hofstätter, Kirsten Falk, Olaf Rötzschke

**Affiliations:** 1 Max-Delbrück-Center for Molecular Medicine (MDC), Berlin, Germany; 2 SIgN (Singapore Immunology Network), A*STAR (Agency for Science, Technology and Research) Singapore, Singapore; University of Lyon, France

## Abstract

**Background:**

Autoimmune diseases result from a breakdown in self-tolerance to autoantigens. Self-tolerance is induced and sustained by central and peripheral mechanisms intended to deviate harmful immune responses and to maintain homeostasis, where regulatory T cells play a crucial role. The use of self-antigens in the study and treatment of a range of autoimmune diseases has been widely described; however, the mechanisms underlying the induced protection by these means are unclear. This study shows that protection of experimental autoimmune disease induced by T cell self-epitopes in a multimerized form (oligomers) is mediated by the induction of active suppression.

**Principal Findings:**

The experimental autoimmune encephalomyelitis (EAE) animal model for multiple sclerosis was used to study the mechanisms of protection induced by the treatment of oligomerized T cell epitope of myelin proteolipid protein (PLP_139–151_). Disease protection attained by the administration of oligomers was shown to be antigen specific and effective in both prevention and treatment of ongoing EAE. Oligomer mediated tolerance was actively transferred by cells from treated mice into adoptive hosts. The induction of active suppression was correlated with the recruitment of cells in the periphery associated with increased production of IL-10 and reduction of the pro-inflammatory cytokine TNF-α. The role of suppressive cytokines was demonstrated by the reversion of oligomer-induced protection after *in vivo* blocking of either IL-10 or TGF-β cytokines.

**Conclusions:**

This study strongly supports an immunosuppressive role of repeat auto-antigens to control the development of EAE with potential applications in vaccination and antigen specific treatment of autoimmune diseases.

## Introduction

Studies on the prevention and treatment of autoimmune diseases have been focussed to the identification of antigens from pathogens and autoantigens responsible for triggering autoimmune reactions. Under determined conditions, autoantigens able to induce autoimmune disease can also suppress disease in a number of experimental models [1], [2]. Induction of antigen specific tolerance based on the persistence of antigen can be achieved after injection of high-dose antigen or repeated injections of low dose soluble antigens [3], [4], [5]. T cell deletion, anergy mechanisms and active suppression by regulatory cells constitute essential factors in the maintenance of tolerance induced by these means [3], [6]. Active suppression represents one of the dominant mechanisms in the control of autoreactivity characterized by deviation of the immune response via the secretion of suppressive cytokines [7], [8].

The use of multivalent antigens represents an useful approach to deal with the dose/concentration of the antigen required to induce tolerance [9], [10], [11]. As an example, the synthetic repetitive Copolymer-1 (Glatiramer Acetate), approved as a therapy for relapsing remitting multiple sclerosis [12], contains sequences that cross react with the myelin basic protein [13], [14] and produce immunomodulatory effects involving the induction of specific T suppressor cells and bystander suppression mechanisms [15]. Despite several therapies for multiple sclerosis exist, their efficacy is very limited and most of the drugs slow the progression of the disease and reduce the number of relapses, however no complete cure is achieved [16], [17].

Our previous studies describe the role of repetitive oligomerized peptides in the control of autoimmune diseases. It was shown that a single low dose injection of oligomers, consisting of repeats of an encephalitogenic T cell epitope from the proteolipid protein of myelin, controlled the development of EAE in mice [9] and oligomers of the neuritogenic epitope of myelin P2 protein prevented the induction of experimental autoimmune neuritis (EAN) [10]. Likewise, multimerized self epitopes in the type I diabetes model demonstrated to provide protection against the disease and it was correlated with the expansion of FoxP3^+^ regulatory cells [11].

Oligomers have proved to be effective in inducing strong immune response because of their ability to crosslink efficiently class II molecules of the major histocompatibilty complex (MHC-II) and to trigger signalling through the T cell receptor (TCR). This might result in improved antigenicity due to the activation of antigen presenting cells [18] and increased T cell proliferation [19]. The mechanisms of action of oligomerized peptides in suppressing the progression of autoimmune diseases are not completely elucidated. Their suppressive effect has been explained by the induction of anergy [20] or the expansion of regulatory cells [11]. Some of the mechanisms underlying the tolerogenic capacity of repeat antigens are described in this study. The ability of oligomer peptides containing self-antigens to control the development as well as the progression of ongoing experimental autoimmune disease was correlated with the induction of protective tolerance mainly mediated by suppressive cytokines.

## Materials and Methods

### Ethics Statement

The animals were maintained and handled according to the Directive 86/609/ECC of the European Community Council and to the institutional, state and federal guidelines. All animal experiments were approved by the Landesamt für Arbeitsschutz, Gesundheitsschutz und Technische Sicherheit (Berlin, Germany). Animals were housed under standard conditions of 12-hour light/dark cycle and given access to food and water *ad libitum*. During periods of active paralysis, animals were provided gel pack and/or moistened food on the cage floor. Animal suffering was reduced by keeping the number of animals used to a minimum required for the planned studies to give statistically significant data. Mice were independently observed and monitored daily by both researchers and animal care staff. Animals were euthanized when they met the following humane endpoint criteria: i) animals with hind-limb and fore-limb paralysis for more than three days without indication of weight-gain and ii) any animal developing loss of bladder control for more than three days. Animals were sacrificed once the experiment was completed. In all cases, mice were humanely sacrificed by injecting an overdose of an anaesthetic agent (Ketamine/Rompun) solution.

### Animals

10 to 12 weeks old, female SJL/J mice were used throughout the experiments. Mice were purchased from Taconic Laboratories (Rey, Denmark) or Charles River Laboratories (Sulzfeld, Germany) and maintained under specific pathogen free conditions in the animal facility at the Max Delbrück Center.

### Antigens

Proteolipid protein derived PLP_139–151_ (C140S) ((HSLGKWLGHPDKF) later referred as PLP_139–151_ (Research Genetics EMC), was synthesized by using standard solid phase F-moc chemistry. All peptides were purified on a C4 -HPLC column (Vydac). Oligomerized T cell epitopes, PLP_139–151_ 16-mer (oligomer containing 16 repeats of the encephalitogenic epitope derived from the proteolipid protein PLP), were produced in *Escherichia coli* bacteria using recombinant techniques as described [18]. In brief, double-stranded oligonucleotide units encoding the T cell epitopes of the PLP_139–151_ (C140S) oligomers were generated by annealing two complementary strands of synthetic oligonucleotides (PLP_139–151_ (C140S), +strand: 5′-TCACTCTCTGGGTAAATGGCTGGGTCACCCGGATAAATTCGG, and - strand: 5′-GAATTT-ATCCGGGTGACCCAGCCATTTACCCAGAGAGTGACC). They were linked to the nucleotide sequence of the S3 spacer GGPGGGPGGGPGG by cloning the oligonucleotides into the BsrDI site of a modified pCITE vector (Novagen), which contained the DNA encoding the S3 spacer as described [9], [18]. The HA_107–119_ 4-mer consists of four covalently linked linear HA_107–119_ peptides (SVSSFERFEIFPK), each linked by the S3 spacer. All oligomers were produced in *E. coli* using recombinant techniques as previously described [18]. Endotoxin was removed from the polypeptide oligomers by separation on a reversed-phase C4-HPLC column (Vydac) and tested for endotoxin with the colorimetric limulus test (Charles River Laboratories).

### Antibodies

For in *vivo* injection, mouse monoclonal antibodies (mAb), were produced from hybridoma cell lines at the Max Delbrück Center. All hybridoma cell lines were kindly provided by Prof. Alexander Scheffold, Deutsches Rheuma-Forschungszentrum Berlin, Germany (DRFZ): anti-interleukin-10 (α-IL-10) (clone JES5-2A5) [21], anti-IL-10 receptor (α-IL-10R) (clone 1B1.3a) [22], anti-transforming growth factor beta (α-TGF-β) (clone 1D11) [23], anti-interleukin-4 (α-IL4) (clone 11B11) [24], α-CD25 (clone PC61) [25]. Rat IgG1 isotype (clone G1-113) was obtained from Sigma. Briefly, antibodies were harvested from culture supernatants and precipitated with ammonium sulphate. Afterwards they were purified over Protein G columns (Thermo scientific-Pierce, Germany) and dialysed against phosphate buffered saline (PBS). The protein concentration was determined by Bradford test and the endotoxin level was measured with the limulus method. Endotoxin levels were <1 EU/mg protein. For flow cytometry staining: α-CD4-PerCP-Cy5.5 (clone RM4-5) and anti-tumor necrosis alpha (TNF- α) APC (clone XT22) were from BD Pharmingen (San Diego, CA). α-CD154-PE (clone MR1) was purchased from Miltenyi Biotech (Bergisch-Gladbach, Germany). For separation of cell subpopulations: α-CD43-PE (clone L11) was obtained from Miltenyi Biotec. α-CD45R/B220-PE (clone RA3-6B2), α-CD25-FITC (clone PC61) and α-PE/FITC microbeads were purchased from BD Pharmingen.

### EAE induction and treatment

Mice were immunized subcutaneously at the base of the neck and tail with 50 µg of PLP_139–151_ (C140S) peptide in incomplete Freund's adjuvant (IFA) (Sigma) containing 400 µg of *Mycobacterium tuberculosis* H37Ra (Difco Laboratories- Detroit USA). One day after priming, mice were injected intravenously with 200 ng of *Pertussis* toxin (List Biological Laboratories, Inc UK) and scored daily for clinical signs of EAE, according to a standard scale to monitor the severity of EAE course [26]. The clinical score was assessed as follows: 0, no clinical sign; 1, flaccid tail; 2, flaccid tail and impaired righting reflex: 3, partial hind limb paralysis; 4, hind and forelimb paralysis; 5, moribund. The effect of the PLP_139–151_ 16-mer peptide in the progression of EAE was tested before and after induction of the disease. Mice were vaccinated 7 days before or were treated 7 days after EAE induction. The therapeutic effect of oligomers was also tested after the appearance of the first clinical signs of disease. In every case, 50 µg of PLP_139–151_ 16-mer peptide dissolved in PBS was administered intravenously. For vaccination experiments, two routes of injection were tested; intravenous injection and subcutaneous injection in IFA. To test the antigen-specific effect of the oligomer, 50 µg of an unrelated oligomer peptide HA_107–119_ 4-mer was used for the EAE treatment.

### T cell proliferation assay

Spleen and lymph node cells from the EAE control group and from oligomer treated mice were isolated on day 13 after disease induction. Pooled spleen and lymph node cells were cultured at a density of 3×10^5^ cells per well in 96-well plates in RPMI-1640 medium (Invitrogen- Karlsruhe, Germany) supplemented with 5% fetal calf serum (FCS) (Invitrogen). Cells were stimulated with titrated amounts of PLP_139–151_ peptide and after 72 hours incubation, the cultures were pulsed with 1 µCi (1 Ci = 37 GBq) of ^3^H-thymidine (Amersham, Freiburg Germany) and incubated for additional 16 hours. Cells were harvested for scintillation counting in a Microplate Counter (Wallac 1420 Victor3TM-Turku, Finland). Stimulation index was calculated by dividing the PLP_139–151_ induced proliferation by proliferation in cultures with medium only.

### Cytokine production

To measure cytokine production, a single suspension of pooled spleen and lymph node cells were used. For IL-10 detection, 4×10^5^ cells were stimulated with 10 µg/ml PLP_139–151_ monomer peptide or PLP 16-mer in RPMI-1640 10% FCS for five days at 37°C. Cultures were re-stimulated with the antigen and irradiated autologous antigen presenting cells for additional five days. IL-10 levels in the supernatants were quantified by ELISA using mouse-IL-10 (OptEIA ™, BD Biosciences). Briefly, plates were coated with IL-10 capture antibody in carbonate buffer and blocked with a PBS-10% FCS for 1 hour at room temperature. Supernatants were added to the wells and incubated for 1 hour at room temperature. Plates were further incubated with biotinylated anti-mouse IL-10 and avidin-horseradish peroxidase conjugate and developed by the addition of TMB substrate solution (KPL Gaithersburg, Maryland USA). IL-10 levels in culture supernatants were determined by interpolation from the IL-10 standard curve. Reactions were measured at 450 nm absorbance in an ELISA reader (VictoR 3V TM Perkin Elmer- Germany).

For TNF-α detection, 5×10^5^ cells were isolated from oligomer treated (13 days after EAE induction) and untreated mice. Cells were activated for 6 hours with 20 µg/ml PLP_139–151_ peptide and 4 µg/ml α-CD28 (clone 37.51) [27] produced at the Max Delbrück Center (the hybridoma cell line was provided by Prof. Alexander Scheffold) To block cytokine secretion, 5 µg/ml Brefeldin A (Sigma) was added for the last four hours of stimulation. Cells were resuspended in PBS-2% FCS/EDTA 2 mM (MACS buffer) and stained for surface CD4, followed by fixation and permeabilization for 30 minutes at 4°C using the Cytofix-Cytoperm solution (BD Biosciences Pharmingen). For detection of cytokines in antigen-specifically activated T cells, combined intracellular CD154 and TNF-α staining was performed [28]. The frequency of CD154^+^ TNF-α^+^ double positive cells in the total CD4^+^ lymphocytes was determined and analysed on a LSR II cytometer with a BD Bioscience FACSDivaTM software.

### Adoptive cells transfer

Spleen cells and lymph node from oligomer treated and non-treated mice were collected 13 days after EAE induction. Cell suspensions were prepared by mincing tissue fragments in medium and filtered through a nylon mesh (BD-Falcon). Erythrocytes were lysed with a hypotonic ammonium chloride solution (NH_4_Cl 0.83%) for 3 minutes at 37°C. Total lymphoid cells and different cell subpopulations were assessed for the ability to confer protection to recipient mice in adoptive transfer experiments. Subpopulations of B220^+^, CD4^+^CD25^+^ and CD4^+^CD25^−^ cells were separated by immunomagnetic cell sorting. Briefly, 20 µl of α-CD43 and 10 µl of microbeads were added per 1×10^7^ total cells and incubated for 20 min at 4°C. Untouched B cells were collected in the column effluent using MidiMACS LD columns (Miltenyi Biotech). Positive and negative selection was used to isolate CD4^+^CD25^+^ and CD4^+^CD25^−^ cells. In brief, B cells were depleted using α-CD45R/B220 and microbeads. The fraction of B220^−^ cells was incubated with α-CD25 and microbeads and after magnetic separation, CD4^+^CD25^+^ cells were enriched using MidiMACS LScolumns (Miltenyi Biotech). The negative fraction was enriched in CD4^+^CD25^−^. Purity of isolated cell populations was greater than 95% as determined by flow cytometry. 5×10^6^ total lymphoid cells or 5×10^6^ cells from each subpopulation were resuspended in PBS 1% FCS and intravenously transferred to recipient mice, one day before the EAE induction.

### In vivo neutralization of cytokines

mAb α-IL-10R was used to block IL-10 signalling. mAbs α-IL-10, α-TGF-β, α-IL-4 and IgG1 isotype control, were used for *in vivo* neutralization of cytokines. PLP_139–151_ 16-mer treated mice were injected intraperitoneally on days 7, 9, 11 and 13 after disease induction with 0.5 mg of the corresponding antibody. The effect of cytokine blockade on the clinical course of EAE was monitored daily.

### Statistical analysis

For comparison of clinical EAE scores, significance between the groups was determined by nonparametric Mann-Whitney U test. Data represent the mean ±SEM ***P<0.001, **P<0.01, *P<0.05. Statistical differences in proliferation and cytokine responses were evaluated using Student's *t* test. P values *P<0.05 were considered significant. Sigmaplot software (San Jose).

## Results

### Prevention of EAE induced by administration of PLP_139–151_ 16-mer and antigen specificity

**Figure 1:**
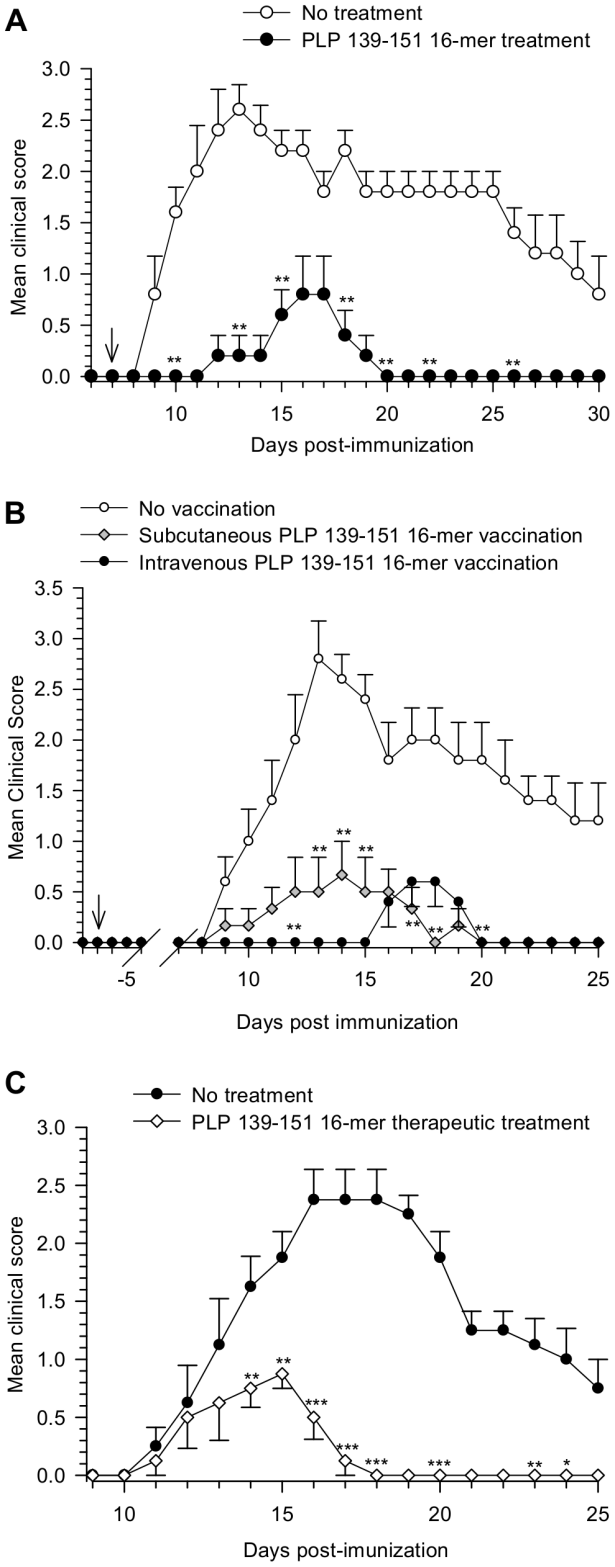
Protection against EAE development after administration of PLP_139–151_ 16-mer. (A) EAE was induced in SJL/J mice with 50 µg of the encephalitogenic PLP_139–151_ peptide emulsified in complete Freund's adjuvant containing *Mycobacterium tuberculosis*. Mice were untreated or treated intravenously with 50 µg of the PLP_139–151_ 16-mer on day 7 after disease induction as represented by the arrow. (B) Mice were vaccinated subcutaneously or intravenously with 50 µg of PLP_139–151_ 16-mer. Vaccination was performed 7 days before EAE induction as indicated by the arrow. Subcutaneous injection was given in incomplete adjuvant and for the intravenous injection the oligomer was dissolved in PBS. (C) Mice (n = 8) were treated individually once the first clinical signs of EAE appeared. 50 µg of the PLP_139–151_ 16-mer was given intravenously in diseased mice. The plots show the mean ±SEM daily clinical score. Statistical significance between groups was determined was determined by Mann-Whitney U test. ***P<0.001, **P<0.01 and *P<0.05 compared with respective control group.

We had shown earlier the therapeutic efficacy of multimerized epitopes in the treatment of autoimmune diseases [9], [10], [11]. In the present study, we tested whether the oligomers were effective before disease induction or when administered after the onset of clinical signs of EAE. We used the susceptible SJL/J (H-2^s^) strain of mice to evaluate the effect of the epitope oligomer on the evolution of EAE. Upon immunization with the PLP_139–151_ epitope, mice developed clinical signs of EAE around day 10, reaching the peak of severity at about 15 days post-induction. The administration of the oligomer PLP_139–151_ 16-mer after or prior EAE induction inhibited the evolution of disease almost completely (Fig. 1A and B). At the peak of clinical disease (day 13), treated and vaccinated mice had a significant decrease in the severity of the disease. Oligomer treated mice showed a mean clinical score of 0.2±0.2, compared with a score of 2.6±0.2 in the control untreated group, p = 0.008 (Fig. 1A). Likewise, epitope-oligomer vaccination of either subcutaneous or intravenous injection routs prevented the EAE development. There was a significant decrease in the severity of the disease in both vaccinated groups, with a mean clinical score of 0.5±0.3 for subcutaneous and 0 for intravenous vaccination compared with a score of 2.8±0.3 in the control group; p = 0.0096 and p = 0.008 respectively (Fig. 1B). To determine the therapeutic effect of the epitope oligomers during disease, mice were given individually PLP_139–151_ 16-mer after the onset of the disease. Figure 1C, shows the effectiveness of the treatment even if delayed until the appearance of the first disease signs. Treated mice showed a mean clinical score of 2.3±0.2 at the peak of disease (day 17) compared with 0.13±0.1 in untreated mice, p = 0.0009.

**Figure 2:**
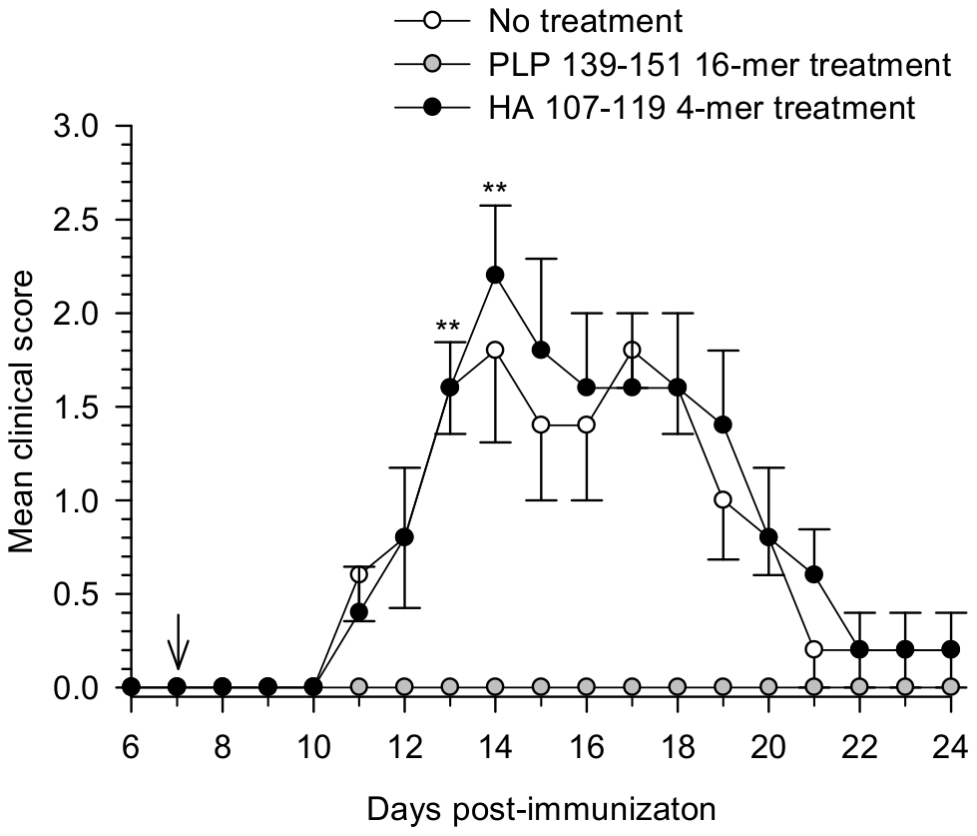
Antigen specificity is required for the protection induced by PLP_139–151_ 16-mer. EAE was induced in SJL/J mice with the PLP_139–151_ peptide in complete adjuvant. On day 7 after disease induction, mice were treated intravenously with PLP_139–151_ 16-mer or with an unrelated oligomer containing an epitope derived of the influenza virus hemagglutinin protein (HA_107–119_ 4-mer) as indicated by the arrow. Mice were monitored daily and the average ±SEM of the clinical score of five mice per group was calculated. Statistical significant difference between the PLP_139–151_ 16-mer and HA_107–119_ 4-mer treated groups was observed (**P<0.01).

To establish that the protection induced by oligomer treatment was antigen specific, the effect on the suppression of the disease was tested by the use of irrelevant oligomers (4-mers) of an epitope derived from the influenza virus hemagglutinin protein (HA_107–119_) containing the same spacer sequence linked to the NH2-terminal side (S3 spacer) as the oligomerized encephalitogenic antigen PLP_139–151_ 16-mer. The results show that no protection was achieved in mice treated with the HA_107–119_ 4-mer, as they exhibited a mean maximal clinical score of 2.2±0.3, comparable with 1.8±0.4 in untreated mice with disease incidence of 100% for both groups. On the other hand, mice treated with PLP_139–151_ 16-mer were completely protected against the disease (Fig. 2). The plot shows a significant difference (p = 0.008) on the protection conferred by the oligomer containing the specific antigen compared to the unrelated oligomer.

### *Ex vivo* proliferation assay

**Figure 3:**
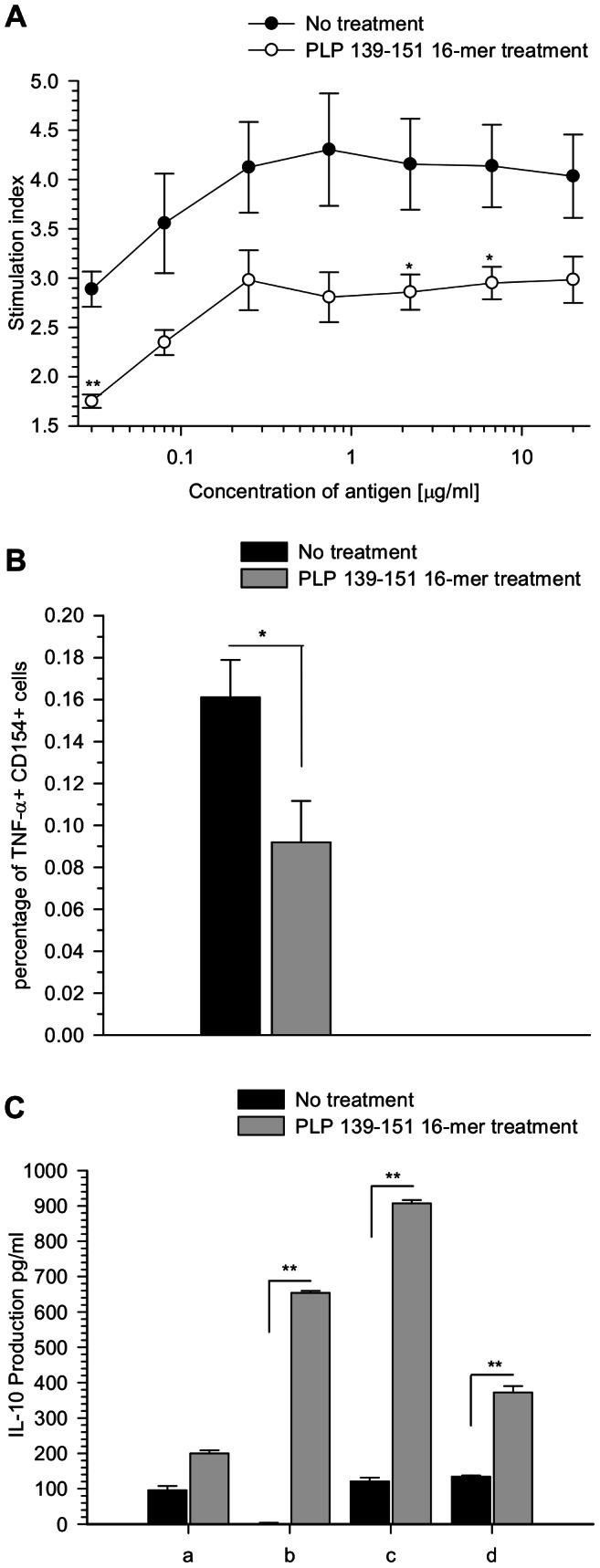
Antigen specific stimulation and cytokine production of cells from PLP_139–151_ 16-mer treated mice. (A) Lymphoid tissue cells from oligomer treated mice were isolated on day 13 post-immunization and stimulated with titrated amounts of PLP_139–151_ peptide. 3×10^5^ cells were incubated for 72 hours and the T cell response was measured by ^3^H-thymidine incorporation. Stimulation index was calculated as the ratio of mean counts per minute (cpm) from quadruplicate mice to the mean background cpm. An index >2 was considered positive. **P<0.01 and *P<0.05 compared to untreated controls by Student's *t* test analysis. (B) *Ex vivo* TNF-α secretion by cells from PLP_139–151_ 16-mer treated and untreated mice was measured. Cells were isolated on day 6 after oligomer treatment and stimulated with 20 µg/ml PLP_139–151_ peptide and 4 µg/ml α-CD28 for 6 h. The intracellular TNF-α cytokine was quantified on CD4^+^CD154^+^ activated cells by FACS analysis. *P<0.05 compared to untreated controls by Student's *t* test analysis. (C) To measure IL-10 secretion, lymphoid tissue cells from PLP_139–151_ 16-mer treated mice and untreated mice were stimulated on days 0 and 5 with different combinations of the PLP_139–151_ peptide (monomer) or the PLP_139–151_ 16-mer (oligomer). Stimulation was performed with: (a) monomer-monomer, (b) monomer-oligomer, (c) oligomer-monomer and (d) oligomer-oligomer peptides. After 10 days in culture, supernatants were collected and the production of IL-10 was measured by ELISA. Results are expressed as the mean cytokine concentration (pg/ml). **P<0.01 compared to untreated controls by Student's *t* test analysis.

The effectiveness of antigen specific therapies for autoimmune diseases has been correlated with the lack of antigen T cell response [29]. The *ex vivo* capacity of cells from oligomer treated mice to respond against the cognate antigen was tested. Cells from oligomer treated and untreated mice were isolated on day 13 post-immunization and were challenged *in vitro* with different dosages of PLP_139–151_ peptide. Antigen specific response was not completely inhibited in oligomer treated mice, although a substantial reduction in proliferation was observed (Fig. 3A). On the other hand, significant higher levels of antibodies to the specific peptide were detected in serum of oligomer treated mice compared to the untreated animals (Figure S1).

### Cytokine production

To investigate whether the PLP_139–151_ 16-mer treatment regulates the secretion of pro-inflammatory and anti-inflammatory cytokines, cells from lymphoid tissues of treated and untreated mice were isolated on day 13 post-immunization (6 days after treatment) and the production of TNF-α and IL-10 was measured upon *ex-vivo* stimulation. Intracellular expression of TNF-α was quantified by flow cytometry. Co-expression of CD154 was used as a marker to identify activated antigen specific cytokine-secreting CD4^+^ cells [28]. Figure 3B, indicates the percentage of positive cells for TNF-α and CD154 expression in CD4^+^ cells after antigen stimulation. Stimulation with PLP_139–151_ peptide and α-CD28 revealed increased percentages of TNF-α expressing cells in untreated mice in comparison with treated mice, being 0.16% and 0.09% respectively. Similar results were observed on days 2 and 4 after oligomer treatment (data not shown). Significance differences were *p<0.05 in both CD154 and TNF-α expression.

The anti-inflammatory cytokine IL-10 has been showed to play a pivotal role in tolerance induction; particularly in the EAE model, it has been characterised as a key cytokine in the suppression of the disease [30]. It was of interest to look whether PLP_139–151_ 16-mer treatment could modulate the IL-10 production in comparison to untreated mice. As IL-10 is difficult to detect by intracellular staining methods due to its rapid secretion and short half-life [31], [32], in this study the IL-10 secretion was quantified by ELISA using short-term cell cultures generated after repeated antigen specific stimulation. Combinations of monomeric PLP_139–151_ and oligomer 16-mer peptide for cell stimulation were compared in their ability to modulate the IL-10 production (Fig. 3C).

Results demonstrated enhanced IL-10 production in cultured cells from oligomer treated mice and reduced IL-10 secretion in cells from untreated mice, regardless of the peptide used for the stimulation. In particular, monomer-oligomer stimulation resulted in about 6-fold increase in IL-10 production in treated mice. Similar results were found when IL-10 levels were measured in purified B cells from treated and untreated mice (data not shown).

### Adoptive cell transfer

T and B lymphocytes with regulatory functions have shown to play an important role in the maintenance of peripheral tolerance in autoimmune diseases [33], [34]. The potential suppressive function of cells from oligomer treated mice was tested by adoptive transfer of lymphoid cells and cell populations into EAE-induced SJL/J recipient mice. Modulation of disease severity was assessed by the transfer of purified CD4^+^CD25^+^, CD4^+^CD25^−^ and B cells from oligomer treated and untreated mice.

Protection induced in oligomer treated mice was actively transferred. Mice receiving total spleen cells from the treated group had decreased clinical signs of disease, displaying a mean maximal score of 2.5±1.2. In contrast, mice that received CFA-primed cells developed a normal form of EAE (mean maximal score of 4.2±0.7, p = 0.2) with a relapsing phase at a later time point of the disease course (Table 1). In line with this result, distinct cell subpopulations derived from oligomer treated animals were also effective in reducing the severity of EAE as evidenced by reduction of clinical score and disease incidence. Transfer of CD4^+^CD25^+^, CD4^+^CD25^−^ or B cells from treated mice was correlated with reduced clinical scores, contrary to the transfer of cells from untreated mice, which failed to transfer suppression (Table 1). In particular, mice receiving CD4^+^CD25^+^ or B cells from oligomer treated mice manifested reduced severity of paralysis showing a mean maximal clinical score of 1.7±0.6 and 1.4±0.6 respectively in comparison with 3.0±1.2 (p = 0.4) and 3.0±0.3 (p = 0.056) in their respective control group. Significant differences between the EAE control group and animals transferred with total cells or different cell populations from treated mice were found at the peak of clinical disease (data not shown).

**Table 1:** Adoptive transfer of cells from PLP_139–151_ 16-mer treated mice.

Transfer	Donor	No. EAE	Mean Group Score ±SEM^a^	Mean EAE Score ±SEM^b^	Mean Day of Onset ±SEM
B220^+^ cells	Oligomer treated mice	3/5	1.4±0.6	2.3±0.3	13.3±1.4
	Untreated mice	4/5	2.8±0.8	3.5±0.5	12.2±0.6
	EAE Control – No transfer	5/5	3.0±0.3	3.0±0.3	12.2±1.0
CD4^+^CD25^+^	Oligomer treated mice	3/4	1.7±0.6	2.3±0.3	8.3±0.3
CD4^+^CD25^−^	Oligomer treated mice	4/4	2.5±0.3	2.5±0.3	13.5±2.6
	EAE Control – No transfer	3/4	3.0±1.2	4.0±1.0	13.7±1.2
Spleen cells	Oligomer treated mice	3/4	2.5±1.2	3.3±1.2	10.0±1.0
	CFA primed mice	4/4	4.2±0.7	4.2±0.7	11.7±0.7

Subpopulations of B220^+^, CD4^+^CD25^+^ and CD4^+^CD25^−^ cells were purified from CFA primed mice and oligomer treated and untreated SJL/J mice. Cell separation was performed by immunomagnetic sorting. 5×10^6^ total spleen cells or 5×10^6^ cells from each subpopulation were resuspended in PBS 1% FCS and intravenously injected into recipient mice, one day before the EAE induction. The effect of adoptive cell transfer to modulate the evolution of EAE was assessed. Animals were monitored daily for development of EAE.

a Mean ± SEM of maximum clinical score of EAE from all animals in the group.

b Mean ± SEM of maximum clinical score from animals exhibiting EAE within a group.

### Neutralization of anti-inflammatory cytokines influence the suppressive effect induced by oligomer treatment

**Figure 4:**
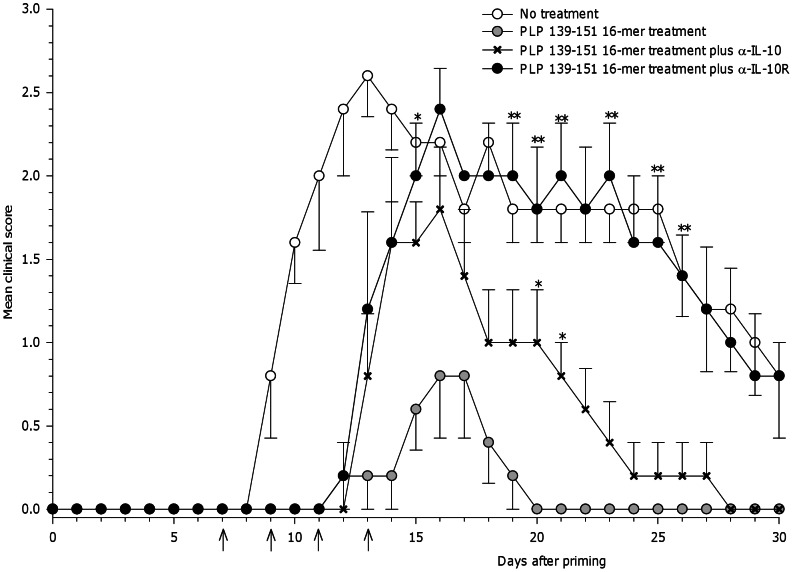
Effect of *in vivo* neutralization of IL-10 on the PLP_139–151_ 16-mer treatment. Oligomer treated mice were intraperitoneally injected with 0.5 mg of α-IL-10 or α-IL-10R (anti-IL-10 receptor). Injections were performed four times from the day of the treatment, every two days as indicated by the arrows. Untreated mice were used as control. Mice were monitored daily and the mean clinical score ±SEM was analyzed. These results are representative of two independent experiments. Statistical significance, **P<0.01 and *P<0.05 compared to PLP_139–151_ 16-mer treated mice that were not administered anti-cytokine blocking antibodies. No effect was observed in oligomer treated mice given isotype control IgG1 antibody (Figure S2).

Previous studies have shown that IL-10 and TGF-β have anti-inflammatory and protective effects in a variety of autoimmune diseases [35], [36], [37]. To test whether IL-10 *in vitro* production observed in treated mice directly correlates to the protective effect provided by the oligomer treatment *in vivo*, mice were systemically administered α-IL-10 or α-IL-10 receptor (IL-10R) monoclonal antibodies. The results show that inhibition of IL-10 reverses oligomer-mediated protection of EAE (Fig. 4).

*In vivo* neutralization of IL-10 with either α-IL-10 or α-IL-10R significantly affected the immunomodulatory function of the treatment with PLP_139–151_ 16-mer. More effective blockage of IL-10 was achieved with α-IL-10R antibody than with α-IL-10, as has been reported before [38]. Analysis of the clinical EAE scores shows that oligomer treated mice displayed a peak of relapsing following administration of α-IL-10R antibody on day 15, reaching similar clinical scores as the untreated animals, mean score of 2.0±0.3 and 2.2±0.2 respectively. In contrast, oligomer treated mice that did not receive IL-10 blocking antibodies had significantly decreased EAE signs (mean score of 0.6±0.2) compared to treated animals injected with α-IL-10R (mean clinical score 2.0±0.3, p = 0.016) or α-IL-10 (mean clinical score 1.6±0.2, p = 0.056) (Fig. 4). Injection of isotype control antibody had no effect on the protective effect induced by the treatment (Figure S2).

**Figure 5:**
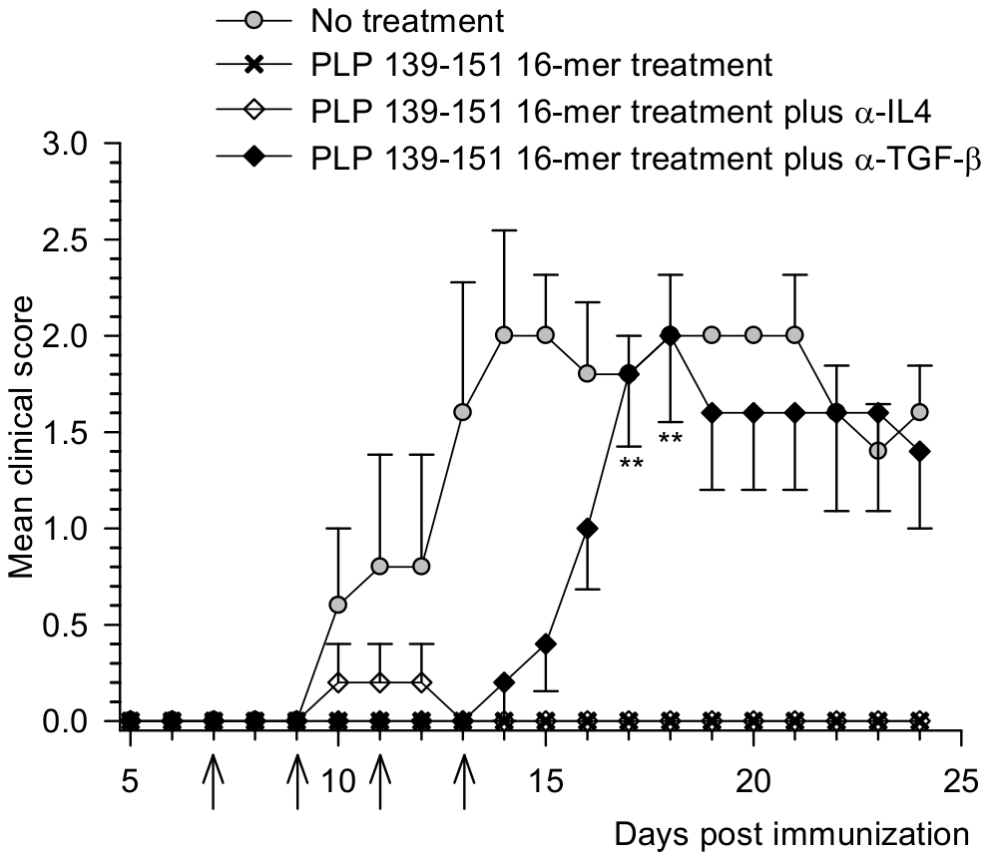
Effect of *in vivo* neutralization of TGF-β on the PLP_139–151_ 16-mer treatment. Oligomer treated mice were intraperitoneally injected with 0.5 mg of α-TGF-β or α-IL4. Injections were performed four times from the day of oligomer treatment every two days as indicated by the arrows. Clinical signs of EAE were scored daily. Data represent the mean ±SEM of the clinical score. **P<0.01 compared to PLP_139–151_ 16-mer treated mice that were not administered anti-cytokine blocking antibodies. No effect was observed in oligomer treated mice injected with isotype control IgG1 antibody (Figure S2).

To identify the role of other suppressive cytokines in the PLP_139–151_ 16-mer induced tolerance to EAE, neutralization of TGF-β was carried out. Administration of anti-TGF-β completely impairs the ability of the PLP_139–151_ 16-mer to inhibit the EAE development. On day 13, all oligomer treated mice receiving TGF-β blocking antibodies presented EAE signs and about day 18 they reached the same score as the untreated mice (mean maximal score of 2±0.4), whereas complete protection was achieved in oligomer treated animals (p = 0.008).

(Fig. 5). Conversely, neutralization of IL-4 did not affect the inhibition of disease development in treated mice. Thus, neutralization of IL-10 and TGF-β was sufficient to abrogate EAE protection in treated mice, suggesting that oligomers play an important role in the modulation of the immune response.

## Discussion

In this study we show that vaccination and therapeutic treatment with PLP_139–151_ 16-mer prevented efficiently the development of EAE. Importantly, the administration of oligomers after the appearance of the first clinical signs of EAE could control the evolution of the disease. The broaden effectiveness of oligomerized peptides to induce tolerance has been described in the context of high-zone tolerance [18] as a threshold in the number of antigen recognition sites associated by repeating antigenic determinants [39], [40].

The protection induced by oligomer T cell epitope PLP_139–151_ was dependent of antigen specificity. An unrelated oligomer HA_107–119_ 4-mer, containing the epitope derived from the influenza virus, which also contains the same spacer sequence as the PLP_139–151_ 16-mer between repeats, did not have effect in the protection of EAE. Antigen-specific therapies that involve T cell recognition for the regulation of autoimmune response represent one of the most reliable approaches to treat or prevent autoimmune disorders [41], [42].

In our previous studies, the histopathological analysis of the brain and spinal cord from oligomer treated mice showed no inflammation or demyelination in contrast to control mice [9]. In line with these observations, here we found significantly less infiltration of cells in the CNS of treated animals and higher numbers of cells were recruited in spleen and lymph nodes. Throughout the experiments performed, increased size of spleen and lymph nodes was observed in treated animals (data not shown). The immunological study of cells from oligomer treated and untreated mice was carried out in cells from peripheral tissues. The phenotypic characteristics of lymphocyte populations isolated from oligomer treated mice did not reveal considerable differences in the expression of markers associated with regulatory functions, co-stimulation, migration markers or activation molecules compared with untreated animals (data not shown).

In addition, oligomer treatment did not induce antigen-specific unresponsiveness. Although to a lesser extend, cells from treated mice proliferated after *in vitro* activation with the cognate peptide. Similar findings have been documented in other experimental models for the treatment of EAE, where normal proliferation against self antigens is achieved in tolerized animals with high doses of antigen, DNA encoding self peptides or recombinant TCR ligands [43], [44]. The analysis of the humoral response reveals that shortly following the treatment with PLP_139–151_ 16-mer, high levels of antigen specific antibodies were produced, although there were not significant differences in the concentration of IgG subclasses. In contrast, untreated mice only produced low amounts of the different isotypes (Figure S1).

Adoptive transfer of serum from treated mice had no significant effect on the evolution of EAE (data not shown). A feature of multivalent antigens is the ability to induce B cells for the production of antibodies with the help of specific CD4^+^ cells [45], [46]. The role of these antibodies and the formation of immune complexes in relation with inhibitory Fc receptors is a subject for future research.

After antigen stimulation *in vitro*, cells derived from oligomer treated mice showed significant reduction in TNF-α secretion and enhanced IL-10 production in contrast to untreated mice. This suggests a role of oligomerized epitopes in the modulation of the inflammatory response by inducing antigen-specific cells to the production of suppressive cytokines. Interestingly, total spleen cells, CD4^+^CD25^+^ and B cells from oligomer treated mice actively transferred protection to recipient mice. Mice transferred with CD4^+^CD25^+^ cells from oligomer treated donors had earlier onset of disease, possibly due to the presence of autoreactive effector CD25^+^ cells in the CD25^+^ regulatory T cell pool. However, these animals recovered in a short time showing significant decrease in the severity of EAE. CD25^−^ cells were less effective in the suppression of disease. The role of CD4^+^CD25^+^ cells in the regulation of autoimmune diseases has been widely described [47]. In future experiments, the DEREG mouse model in which all Foxp3^+^ cells are depleted would be useful to explore the role of T regulatory cells in the oligomer-induced protection [48]. Likewise, adoptive transfer of B cells from oligomer treated mice ameliorated the severity of disease in recipient mice. These cells secrete increased amounts of IL-10 after *in vitro* activation with α-CD40 compared to B cells from the control group (data not shown). This is in agreement with previous reports, which demonstrate that the regulatory role of B cells is mainly mediated by IL-10. Some of these studies indicate that treatment with B cells producing IL-10 can modulate the course of arthritis, inflammatory bowel disease and EAE [34], [49], [50]. *In vivo* IL-10 production is one of the possible mechanisms by which B cells from oligomer treated animals transfer suppression of EAE.

The immunomodulatory role of suppressive cytokines such as IL-10 and TGF-β was found to be crucial for the EAE protection induced by repetitive oligomers. *In vivo* neutralization of IL-10 or TGF-β reversed the protective effect induced by PLP_139–151_ 16-mer treatment. IL-10 and TGF-β are key molecules in the maintenance of peripheral tolerance. According to a number of studies, IL-10 exerts down-modulatory functions that may diminish autoimmune pathologies. IL-10-deficient mice have shown to develop more severe EAE than wild-type mice, and some IL-10 promoter polymorphisms are associated with progression of multiple sclerosis [36], [51]. Additionally, IL-10 has also been implicated in various immunotherapeutic models in which its administration can inhibit the development of EAE [52], [53]. In the same way, TGF-β also plays an important role in T cell homeostasis and prevention of inflammatory autoimmunity [54]. TGF-β deficient mice develop systemic inflammation and abrogation of TGF-β signalling results in spontaneous T cell differentiation and autoimmune disease [55]. The involvement of different subpopulations of T regulatory cells (Tr1 cells) in the production of IL-10 and TGF-β and/or Th3 cells that secrete TGF-β [56], [57] during the oligomer treatment, is an interesting topic for future research.

This study shows that the protection achieved by the oligomer treatment is mainly mediated by the induction of active suppression, which involves the expansion of antigen-specific cells that mainly produce anti-inflammatory cytokines. We added evidence that active suppression underlies the therapeutic efficacy of oligomers in EAE, suggesting that the use of oligomerized-self epitopes as an antigen-specific therapy constitutes an alternative approach in the modulation of the immune response in autoimmune diseases.

## Supporting Information

Figure S1 **Determination of antibody response to PLP_139–151_ in oligomer treated mice.** Mice were treated intravenously with 50 µg of the PLP_139–151_ 16-mer on day 7 after EAE induction. PLP_139–151_-specific antibody determination was tested two weeks after oligomer treatment. Levels of IgG isotypes in serum samples from treated and untreated mice were determined by ELISA. Higher levels of the different isotypes of antibodies specific to the cognate antigen were observed in oligomer treated mice in comparison to the untreated ones.
(TIF)

Figure S2 **Effect of** ***in vivo*** **neutralization of IL-10R on the PLP_139–151_ 16-mer treatment.** Oligomer treated mice were intraperitoneally injected with 0.5 mg of α-IL-10R (anti-IL-10 receptor) or isotype control IgG1 antibody. Injections were performed four times from the day of the treatment, every two days. Oligomer treated mice injected with α-IL-10R developed EAE similar to untreated mice, in contrast to oligomer treated animals injected with the isotype control antibody, which developed very mild disease. Statistical significance between these two groups was observed at the peak of clinical disease (day 14) *P<0.05.
(TIF)
