# Supplementary figures and images for: Active Suppression Induced by Repetitive Self-Epitopes Protects against EAE Development
Source: PLoS One. 2013 May 30;8(5):e64888. doi: 10.1371/journal.pone.0064888 (PMC3667816; doi:10.1371/journal.pone.0064888)

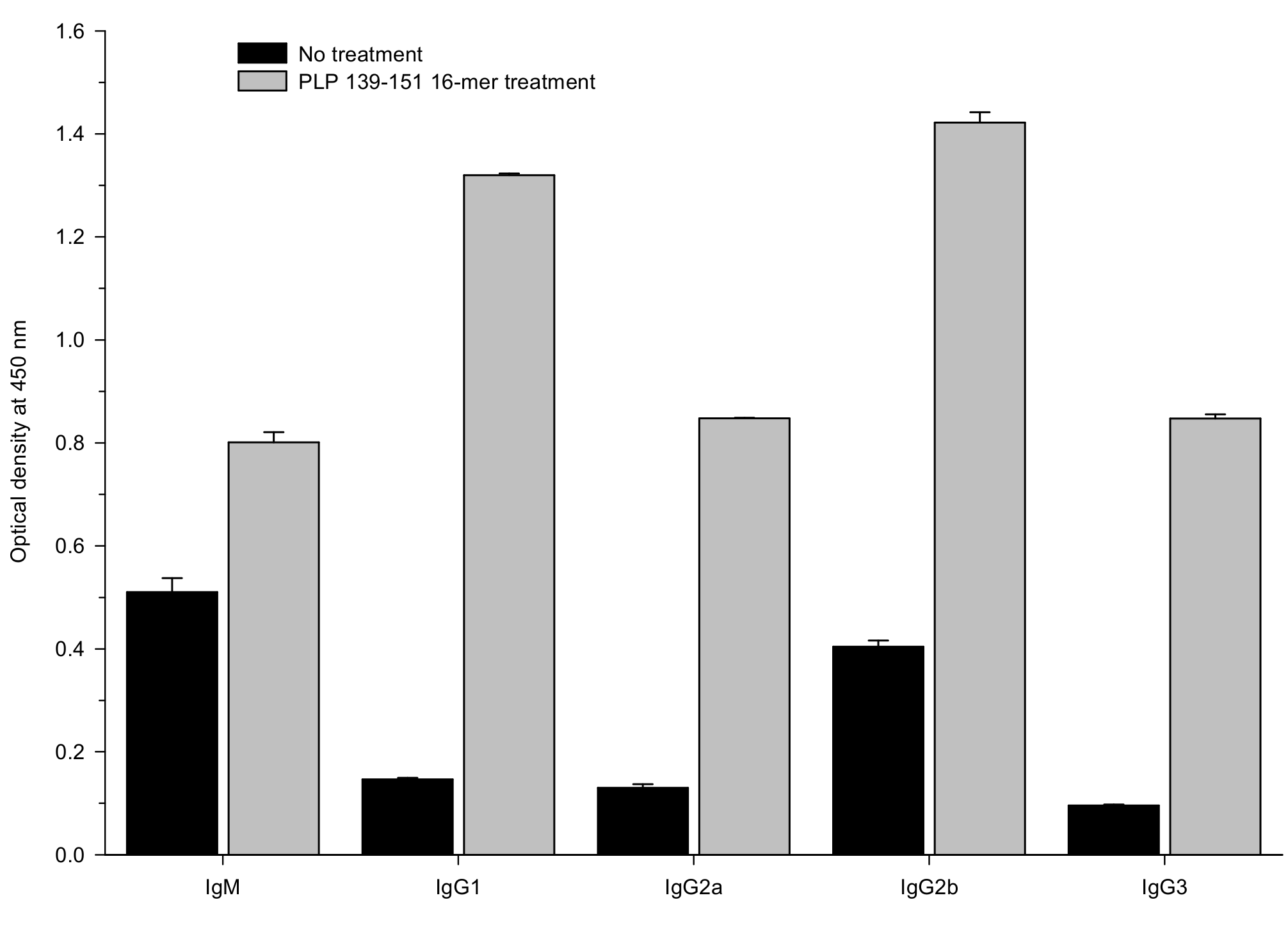

Supplement: Figure S1 — Determination of antibody response to PLP139–151 in oligomer treated mice. Mice were treated intravenously with 50 µg of the PLP139–151 16-mer on day 7 after EAE induction. PLP139–151-specific antibody determination was tested two weeks after oligomer treatment. Levels of IgG isotypes in serum samples from treated and untreated mice were determined by ELISA. Higher levels of the different isotypes of antibodies specific to the cognate antigen were observed in oligomer treated mice in comparison to the untreated ones. (TIF) [file pone.0064888.s001.tif]

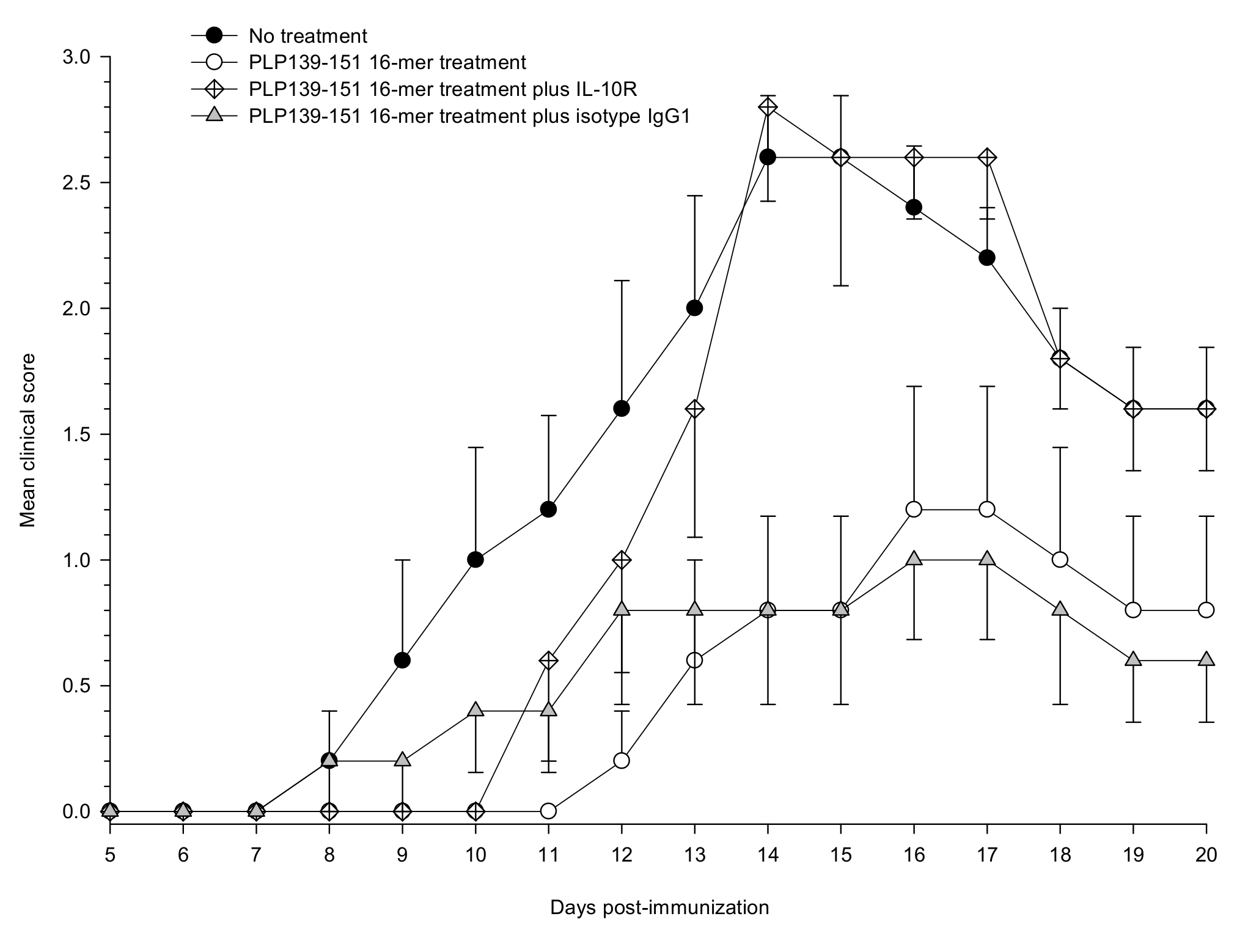

Supplement: Figure S2 — Effect of in vivo neutralization of IL-10R on the PLP139–151 16-mer treatment. Oligomer treated mice were intraperitoneally injected with 0.5 mg of α-IL-10R (anti-IL-10 receptor) or isotype control IgG1 antibody. Injections were performed four times from the day of the treatment, every two days. Oligomer treated mice injected with α-IL-10R developed EAE similar to untreated mice, in contrast to oligomer treated animals injected with the isotype control antibody, which developed very mild disease. Statistical significance between these two groups was observed at the peak of clinical disease (day 14) *P<0.05. (TIF) [file pone.0064888.s002.tif]
